# Prognostic significance of preoperative serum CA125, CA19-9 and CEA in gastric carcinoma

**DOI:** 10.18632/oncotarget.8770

**Published:** 2016-04-16

**Authors:** Wei Wang, Xiao-Long Chen, Shen-Yu Zhao, Yu-Hui Xu, Wei-Han Zhang, Kai Liu, Xin-Zu Chen, Kun Yang, Bo Zhang, Zhi-Xin Chen, Jia-Ping Chen, Zong-Guang Zhou, Jian-Kun Hu

**Affiliations:** ^1^ Department of Gastrointestinal Surgery, West China Hospital, Sichuan University, Chengdu, China; ^2^ Laboratory of Gastric Cancer, State Key Laboratory of Biotherapy/Collaborative Innovation Center of Biotherapy and Cancer Center, West China Hospital, Sichuan University, Chengdu, China; ^3^ West China School of Medicine, Sichuan University, Chengdu, China; ^4^ Laboratory of Digestive Surgery, State Key Laboratory of Biotherapy, West China Hospital, Sichuan University, Chengdu, China

**Keywords:** gastric cancer, tumor marker, CA125, CA19-9, CEA

## Abstract

The prognostic significance of preoperative serum CA125, CA19-9 and CEA in gastric carcinoma (GC) has been widely reported and is still under debate. Here, we evaluated the prognostic significance of preoperative serum CA125, CA19-9 and CEA in patients with GC. 1692 patients with GC who underwent gastrectomy were divided into the training (from January 2005 to December 2011, *n* = 1024) and the validation (from January 2012 to December 2013, *n* = 668) cohorts. Positive groups of CA125 (> 13.72 U/ml), CA19-9 (> 23.36 U/ml) and CEA (> 4.28 ng/ml) were significantly associated with more advanced clinicopathological traits and worse outcomes than that of negative groups (all *P* < 0.01). In Cox regression analysis, tumor size (*P* < 0.001, *P* = 0.005), pTNM stage (*P* < 0.001, *P* < 0.001) and CA125 (*P* = 0.026, *P* = 0.005) were independent prognostic factors both in two cohorts. Nomograms of these two cohorts based on the number of positive serum tumor markers (NPTM) were more accurate in prognostic prediction than TNM stage alone. Our findings suggested that elevated preoperative serum CA125, CA19-9 and CEA were associated with more advanced clinicopathological traits and less favorable outcomes. In addition, CA125 as an independent prognostic factor should be further investigated. Nomogram based on NPTM could accurately predict the prognosis of GC patients.

## INTRODUCTION

Gastric carcinoma (GC) is one of the most common malignant tumors with high mortality worldwide, especially in East Asia, and the five-year survival rate of GC is low [1–3]. Early diagnosis and treatment are the most efficient ways to improve the prognosis. Although examinations, like CT, ultrasound and gastroscopy, may provide much important information of tumor, it is still not sufficient to obtain complete data preoperatively. The detection of preoperative serum tumor markers is routinely used in a variety of tumor patients, since it may prompt the tumor burden and occult metastases of malignancies [4, 5]. However, the clinical application of preoperative serum tumor markers is still under debate.

The commonly used tumor markers for GC patients in our hospital include carbohydrate antigen 125 (CA125), carbohydrate antigen 19-9 (CA19-9) and carcinoembryonic antigen (CEA). CA125 was first detected in ovarian cancer and had been reported to be positive in 80% of ovarian epithelial tumor patients [6]. In addition, the positivity of CA125 is also seen in breast cancer, pancreatic cancer, gastric cancer and some other tumors [7]. CA19-9 is a specific marker of digestive system tumors, especially pancreatic cancer, with high positive rate preoperatively and high negative rate postoperatively [8]. Thus, it is widely used in the diagnosis and monitoring of pancreatic cancer [9]. CEA, a non-specific tumor marker, was originally used as a serum marker for colorectal cancer and could reflect the existence of a variety of tumors, such as pancreatic cancer, lung cancer and gastric cancer [10]. Although the three tumor markers are detected preoperatively, they are hardly used for the diagnosis of GC because of the poor sensitivity and specificity [11, 12]. However, they can be applied in the evaluation of therapeutic effect, monitoring of recurrence and prediction of prognosis [13, 14].

There are amount of studies focused on the relationship between tumor markers and GC, of which only a few studies explored the correlation between preoperative serum tumor markers and clinicopathological traits and survival of GC patients. According to previous studies, there is no doubt that preoperative serum CA19-9 and CEA levels are correlated with TNM stage (based on American Joint Committee on Cancer (AJCC) 7^th^ edition) [15]. As to other clinicopathological traits, including tumor location, tumor size, microscopic type and differentiation grade, there are still no definitive conclusions. In this study, we explored the relationship between preoperative serum tumor markers (CA125, CA19-9 and CEA) and clinicopathological traits and prognostic information of GC patients.

## RESULTS

### The baseline of clinicopathological traits in the training and the validation corhorts

In this study, a total of 1024 GC patients were enrolled in the training cohort, and there were 668 patients in the validation cohort. We first compared the clinicopathological traits and therapy information between the training and the validation cohorts. And the result showed that the main clinical information were similar between the two cohorts, except adjuvant chemotherapy, tumor location, differentiation grade and M stage (Table 1). The training cohort included more M1 patients and less patients with adjuvant chemotherapy than the validation cohort.

**Table 1:** The clinicopathological traits of patients in the training and the validation cohorts

Patient characteristics			Training *n* (%)	Validation*n* (%)	*P*
Gender	Male		702 (68.6)	454 (68.0)	0.799
	Female		322 (31.4)	214 (32.0)	
Age (ys)	<60		564 (55.1)	339 (50.7)	0.081
	≥60		460 (44.9)	329 (49.3)	
Resection pattern	Distal		575 (56.2)	368 (55.1)	0.132
	Total		280 (27.3)	208 (31.1)	
	Proximal		169 (16.5)	92 (13.8)	
Lymphadenectomy	D1/D1+		550 (53.7)	368 (55.1)	0.578
	D2/D2+		474 (46.3)	300 (44.9)	
Chemotherapy	No		649 (63.4)	348 (52.1)	**<0.001**
	Yes		375 (36.6)	320 (47.9)	
Tumor location	Upper		252 (24.6)	187 (28.0)	**0.002**
	Middle		223 (21.8)	97 (14.5)	
	Lower		529 (51.7)	373 (55.8)	
	Whole		20 (2.0)	11 (1.6)	
Tumor size (cm)	≤2		140 (13.7)	81 (12.1)	0.106
	2-5		487 (47.6)	300 (44.9)	
	5-8		308 (30.1)	227 (34.0)	
	>8		89 (8.7)	60 (9.0)	
Macroscopic type	Type-0		161 (15.7)	90 (13.5)	0.050
	Type-1		38 (3.7)	29 (4.3)	
	Type-2		443 (43.3)	273 (40.9)	
	Type-3		333 (32.5)	225 (33.7)	
	Type-4		49 (4.8)	51 (7.6)	
Differentiation grade	Well		17 (1.7)	5 (0.7)	0.001
	Moderate		193 (18.8)	91 (13.6)	
	Poor		814 (79.5)	572 (85.6)	
pT stage	T1		188 (18.4)	122 (18.3)	0.055
	T2		120 (11.7)	87 (13.0)	
	T3		106 (10.4)	106 (15.9)	
	T4		610 (59.6)	353 (52.8)	
pN stage	N0		310 (30.3)	206 (30.8)	0.378
	N1		199 (19.4)	115 (17.2)	
	N2		191 (18.7)	114 (17.1)	
	N3a		216 (21.1)	145 (21.7)	
	N3b		108 (10.5)	88 (13.2)	
M stage	M0		937 (91.5)	629 (94.2)	**0.042**
	M1		87 (8.5)	39 (5.8)	
pTNM stage	I		221 (21.6)	141 (21.1)	0.240
	II		201 (19.6)	151 (22.6)	
	III		515 (50.3)	337 (50.4)	
	IV		87 (8.5)	39 (5.8)	

### Association between preoperative serum tumor markers and clinicopathological traits

The relationship between preoperative serum tumor markers and clinicopathological traits in the training cohort was summarized in Table 2. The positive rates of serum tumor markers were 42.3% for CA125, 20.0% for CA19-9 and 19.2% for CEA. When three tumor markers were combined, the triple-positive rate was only 3.7% (*n* = 38). In univariate analysis, the higher positive rates of CA125, CA19-9 and CEA were significantly associated with larger tumor size, more advanced macroscopic type and pTNM stage (all *P* < 0.05). In the positive group of each tumor marker, the proportions of stage III-IV were 67.9% for CA125 (+), 77.6% for CA19-9 (+) and 77.7% for CEA (+). When three tumor markers were combined, the proportion of stage III-IV rose to 89.5%. As to lymph node metastasis, the proportions of N+ were 74.8% for CA125 (+), 84.9% for CA19-9 (+) and 84.3% for CEA (+), and it rose to 94.7% when three tumor markers were simultaneously positive (Table 3). CA125 and CA19-9 had significantly higher positive rate in female patients than that in male patients (*P* = 0.001 and *P* = 0.009, respectively), while CEA had remarkably higher positive rate in male patients than that in female patients (*P* = 0.027). However, CA19-9 had obviously higher positive rate in older patients than that in younger patients (*P* = 0.043). Similarly, in the validation cohort, the higher positive rates of all three tumor markers were significantly associated with larger tumor size, more advanced macroscopic type and pTNM stage (all *P* < 0.05)(Table 4).

**Table 2:** Correlation between preoperative serum tumor makers and major clinicopathological traits in the training cohort

Patient characteristics			Cases *n* (%)	CA125 (+)*n* (%)	*P*	CA19-9 (+)*n* (%)	*P*	CEA (+)*n* (%)	*P*
Gender	Male		702 (68.6)	272 (38.7)	**0.001**	125 (17.8)	**0.009**	148 (21.1)	**0.027**
	Female		322 (31.4)	161 (50.0)		80 (24.8)		49 (15.2)	
Age (ys)	<60		564 (55.1)	233 (41.3)	0.485	100 (17.7)	**0.043**	99 (17.6)	0.130
	≥60		460 (44.9)	200 (43.5)		105 (22.8)		98 (21.3)	
Tumor location	Upper		252 (24.6)	93 (36.9)	**0.029**	50 (19.8)	**0.012**	61 (24.2)	0.146
	Middle		223 (21.8)	110 (49.3)		56 (25.1)		40 (17.9)	
	Lower		529 (51.7)	219 (41.4)		91 (17.2)		92 (17.4)	
	Whole		20 (2.0)	11 (55.0)		8 (40.0)		4 (20.0)	
Tumor size (cm)	≤2		140 (13.7)	49 (35.0)	**<0.001**	14 (10.0)	**<0.001**	13 (9.3)	**<0.001**
	2-5		487 (47.6)	180 (37.0)		90 (18.5)		83 (17.0)	
	5-8		308 (30.1)	162 (52.6)		76 (24.7)		78 (25.3)	
	>8		89 (8.7)	42 (47.2)		25 (28.1)		23 (25.8)	
Macroscopic type	Type-0		161 (15.7)	53 (32.9)	**0.002**	19 (11.8)	**0.001**	15 (9.3)	**0.012**
	Type-1		38 (3.7)	14 (36.8)		7 (18.4)		9 (23.7)	
	Type-2		443 (43.3)	185 (41.8)		87 (19.6)		90 (20.3)	
	Type-3		333 (32.5)	159 (47.7)		76 (22.8)		73 (21.9)	
	Type-4		49 (4.8)	22 (44.9)		16 (32.7)		10 (20.4)	
Differentiation grade	Well		17 (1.7)	3 (17.6)	**0.016**	0 (0.0)	0.064	2 (11.8)	0.413
	Moderate		193 (18.8)	71 (36.8)		33 (17.1)		43 (22.3)	
	Poor		814 (79.5)	359 (44.1)		172 (21.1)		152 (18.7)	
pT stage	T1		188 (18.4)	60 (31.9)	**<0.001**	19 (10.1)	**<0.001**	14 (7.4)	**<0.001**
	T2		120 (11.7)	40 (33.3)		16 (12.3)		19 (15.8)	
	T3		106 (10.4)	47 (44.3)		13 (12.3)		15 (14.2)	
	T4		610 (59.6)	286 (46.9)		157 (25.7)		149 (24.4)	
pN stage	N0		310 (30.3)	109 (35.2)	**<0.001**	31 (10.0)	**<0.001**	31 (10.0)	**<0.001**
	N1		199 (19.4)	73 (36.7)		39 (19.6)		33 (16.6)	
	N2		191 (18.7)	78 (40.8)		39 (20.4)		42 (22.0)	
	N3a		216 (21.1)	109 (50.5)		55 (25.5)		60 (27.8)	
	N3b		108 (10.5)	64 (59.3)		41 (38.0)		31 (28.7)	
M stage	M0		937 (91.5)	381 (40.7)	**0.001**	173 (18.5)	**<0.001**	167 (17.8)	**<0.001**
	M1		87 (8.5)	52 (59.8)		32 (36.8)		30 (34.5)	
pTNM stage	I		221 (21.6)	69 (31.2)	**<0.001**	22 (10.0)	**<0.001**	19 (8.6)	**<0.001**
	II		201 (19.6)	70 (34.8)		24 (11.9)		25 (12.4)	
	III		515 (50.3)	242 (47.0)		127 (24.7)		123 (23.9)	
	IV		87 (8.5)	52 (59.8)		32 (36.8)		30 (34.5)	

**Table 3:** Correlation between tumor marker positivity and lymph node metastasis and pTNM stage in the training cohort

	Triple-positive	CA125 (+)	CA19-9 (+)	CEA (+)
N0	2 (5.3)	109 (25.2)	31 (15.1)	31 (15.7)
N+	36 (94.7)	324 (74.8)	174 (84.9)	166 (84.3)
P		**0.006**	0.103	0.089
I-II	4 (10.5)	139 (32.1)	46 (22.4)	44 (22.3)
III-IV	34 (89.5)	294 (67.9)	159 (77.6)	153 (77.7)
P		**0.006**	0.095	0.098

**Table 4:** Correlation between preoperative serum tumor makers and major clinicopathological traits in the validation cohort

Patient characteristics			Cases *n* (%)	CA125 (+)*n* (%)	*P*	CA19-9 (+)*n* (%)	*P*	CEA (+)*n* (%)	*P*
Gender	Male		454 (68.0)	171 (37.7)	**0.007**	97 (21.4)	0.278	109 (24.0)	**<0.001**
	Female		214 (32.0)	104 (48.6)		38 (17.8)		24 (11.2)	
Age (ys)	<60		339 (50.7)	142 (41.9)	0.701	69 (20.4)	0.925	58 (17.1)	0.066
	≥60		329 (49.3)	133 (40.4)		66 (20.1)		75 (22.8)	
Tumor location	Upper		187 (28.0)	78 (41.7)	0.374	46 (24.6)	0.293	46 (24.6)	0.122
	Middle		97 (14.5)	47 (48.5)		20 (20.6)		21 (21.6)	
	Lower		373 (55.8)	145 (38.9)		68 (18.2)		63 (16.9)	
	Whole		11 (1.6)	5 (45.5)		1 (9.1)		3 (27.3)	
Tumor size (cm)	≤2		81 (12.1)	24 (29.6)	**<0.001**	7 (8.6)	**0.001**	12 (14.8)	**0.004**
	2-5		300 (44.9)	101 (33.7)		56 (18.7)		47 (15.7)	
	5-8		227 (34.0)	117 (51.5)		56 (24.7)		61 (26.9)	
	>8		60 (9.0)	33 (55.0)		16 (26.7)		13 (21.7)	
Macroscopic type	Type-0		90 (13.5)	21 (23.3)	**<0.001**	7 (7.8)	**0.002**	8 (8.9)	**0.003**
	Type-1		29 (4.3)	16 (55.2)		4 (13.8)		4 (13.8)	
	Type-2		273 (40.9)	108 (39.6)		56 (20.5)		56 (20.5)	
	Type-3		225 (33.7)	98 (35.6)		57 (25.3)		49 (21.8)	
	Type-4		51 (7.6)	32 (62.7)		11 (21.6)		16 (31.4)	
Differentiation grade	Well		5 (0.7)	3 (60.0)	0.452	0 (0.0)	0.131	0 (0.0)	0.944
	Moderate		91 (13.6)	33 (36.3)		14 (15.4)		19 (20.9)	
	Poor		572 (85.6)	239 (41.8)		121 (21.2)		114 (19.9)	
pT stage	T1		122 (18.3)	35 (28.7)	**<0.001**	12 (9.8)	**<0.001**	14 (11.5)	**0.017**
	T2		87 (13.0)	30 (34.5)		14 (16.1)		19 (21.8)	
	T3		106 (15.9)	41 (38.7)		14 (13.2)		19 (17.9)	
	T4		353 (52.8)	169 (47.9)		95 (26.9)		81 (22.9)	
pN stage	N0		206 (30.8)	59 (28.6)	**0.001**	15 (7.3)	**<0.001**	23 (11.2)	**0.001**
	N1		115 (17.2)	56 (48.7)		29 (25.2)		29 (25.2)	
	N2		114 (17.1)	55 (48.2)		24 (21.1)		24 (21.1)	
	N3a		145 (21.7)	61 (42.1)		42 (29.0)		33 (22.8)	
	N3b		88 (13.2)	44 (50.0)		25 (28.4)		24 (27.3)	
M stage	M0		629 (94.2)	252 (40.1)	**0.020**	126 (20.0)	0.646	119 (18.9)	0.010
	M1		39 (5.8)	23 (59.0)		9 (23.1)		14 (35.9)	
pTNM stage	I		141 (21.1)	39 (27.7)	**<0.001**	12 (8.5)	**<0.001**	19 (13.5)	**0.005**
	II		151 (22.6)	54 (35.8)		19 (12.6)		28 (18.5)	
	III		337 (50.4)	159 (47.2)		95 (28.2)		72 (21.4)	
	IV		39 (5.8)	23 (59.0)		9 (23.1)		14 (35.9)	

The multivariate analysis revealed that, in the training cohort, the positive rate of CA19-9 were significantly associated with age (*P* = 0.008). Gender, pT stage and pN stage (all *P* < 0.05) were associated with all three tumor markers. In the validation cohort, gender (*P* = 0.012) and tumor size (*P* < 0.001) were independently related to CA125. pT stage (*P* = 0.019) and pN stage (*P* = 0.001) were independently associated with CA19-9. However, gender (*P* < 0.001), macroscopic type (*P* = 0.022) and pN stage (*P* = 0.019) were independently related to CEA (Table 5).

**Table 5:** Multivariate analysis of preoperative serum tumor makers with clinicopathological traits in the training and the validation cohorts

	CA125 (+)		CA19-9 (+)		CEA (+)	
	OR (95%CI)	*P*	OR (95%CI)	*P*	OR (95%CI)	*P*
**Training cohort**						
Gender	1.614 (1.231-2.115)	**0.001**	1.650 (1.184-2.299)	**0.003**	0.662 (0.461-0.951)	**0.026**
Age	-	-	1.550 (1.124-2.139)	**0.008**	-	-
pT stage	1.138 (1.005-1.289)	**0.041**	1.264 (1.061-1.507)	**0.009**	1.315 (1.099-1.572)	**0.003**
pN stage	1.197 (1.077-1.331)	**0.001**	1.345 (1.180-1.532)	**<0.001**	1.277 (1.120-1.456)	**<0.001**
**Validation cohort**						
Gender	1.538 (1.101-2.148)	**0.012**	-		0.372 (0.229-0.603)	**<0.001**
Tumor size	1.596 (1.310-1.945)	**<0.001**	-		-	
Macroscopic type	-		-		1.279 (1.036-1.580)	**0.022**
pT stage	-		1.289 (1.043-1.592)	**0.019**	-	
pN stage	-		1.288 (1.106-1.501)	**0.001**	1.194 (1.029-1.385)	**0.019**

OR = odds ratio; CI = confidence interval.

### The prognostic significance of preoperative serum tumor markers in the training cohort

Nine hundred and twenty-four patients (924/1024, 90.2%) were followed up and analyzed in prognosis with median survival time of 85.1 (0.3-129.9) months. For all 924 patients, the 1-, 2-, 3- and 4-cumulative overall survival rates were 77%, 67%, 59% and 55%, respectively. For patients in the positive groups of CA125, CA19-9 and CEA, the 3-year survival rates were 51%, 44% and 43%, respectively, compared with 66%, 63% and 63% for patients in the negative groups of these markers, while the rate in patients with three markers simultaneously positive was 29%.

**Figure 1:**
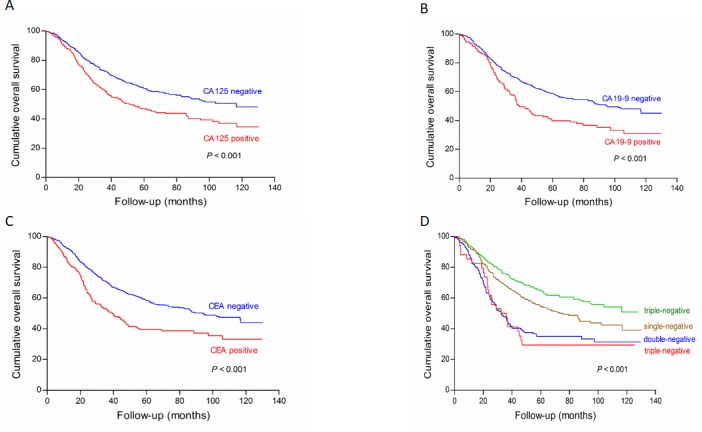
Survival analysis of subgroups of CA125 (A), CA19-9 (B), CEA (C) and their combined detection (D) in the training cohort There were significant differences on survival outcomes between positive and negative subgroups in CA125, CA19-9, CEA and their combined detection, respectively (all *P* < 0.001)

Univariate and multivariate analysis for prognostic factors were shown in Table 6. Compared with the positive groups by Kaplan-Meier analysis, the negative groups of all three tumor markers showed significantly higher survival rates, respectively (Figure 1A, 1B, 1C, all *P* < 0.001). In univariate survival analysis, age (*P* = 0.016), tumor location (*P* < 0.001), tumor size (*P* < 0.001), macroscopic type (*P* < 0.001), differentiation grade (*P* = 0.021), pT stage (*P* < 0.001), pN stage (*P* < 0.001), M stage (*P* < 0.001) and pTNM stage (*P* < 0.001) were significantly associated with prognosis. In a multivariate analysis, age (*P* = 0.011), tumor size (*P* < 0.001), pTNM stage (*P* < 0.001) and CA 125 (*P* = 0.026) were independent prognostic factors.

**Table 6:** Univariate and multivariate analysis of prognostic risk factors for overall survival in the training and the validation cohorts

	Training cohort (*n* = 1024)			Validation cohort (*n* = 668)		
Risk factors	Univariate	Multivariate		Univariate	Multivariate	
	*P* value	HR (95%CI)	*P* value	*P* value	HR (95%CI)	*P* value
Gender	0.401	-	-	0.462	-	-
Age (ys)	**0.016**	1.273(1.057-1.533)	**0.011**	0.590	-	-
Tumor location	**<0.001**	-		**0.001**	-	-
Tumor size (cm)	**<0.001**	1.298(1.142-1.476)	**<0.001**	**<0.001**	1.366(1.100-1.697)	**0.005**
Macroscopic type	**<0.001**	-		**<0.001**	1.280(1.054-1.554)	**0.013**
Differentiation grade	**0.021**	-		**0.022**	-	**-**
pTNM stage	**<0.001**	2.183(1.900-2.508)	**<0.001**	**<0.001**	2.101(1.655-2.668)	**<0.001**
CA125	**<0.001**	1.238(1.026-1.492)	**0.026**	**<0.001**	1.519(1.132-2.039)	**0.005**
CA19-9	**<0.001**	-		**<0.001**	1.431(1.051-1.949)	**0.023**
CEA	**<0.001**	-		**0.001**	-	**-**
NPTM	**<0.001**	1.292(1.040-1.604)	**0.021**	**<0.001**	1.502(1.099-2.052)	**0.011**

HR = hazard ratio; CI = confidence interval; NPTM = number of positive serum tumor markers.

### The prognostic value of nomogram with preoperative serum tumor markers and clinicopathological traits in the training cohort

**Figure 2:**
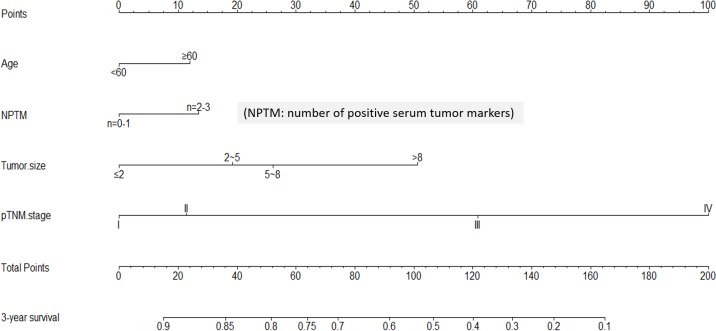
Nomogram of NPTM and clinicopathological traits in the training cohort

**Figure 3:**
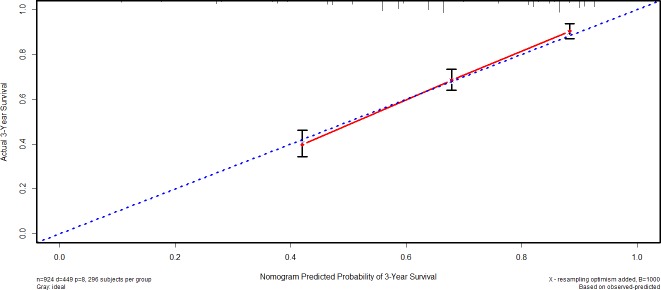
Calibration curve of nomogram in the training cohort

We made a further analysis by using nomogram to predict 3-year overall survival rate of individual patient. When one preoperative serum tumor marker positivity was considered as a single prognostic factor, the number of positive serum tumor markers (NPTM) was likely to be a parameter in nomogram. By using Cox regression test, age (*P* = 0.019), tumor size (*P* < 0.001), pTNM stage (*P* < 0.001) and NPTM (*P* = 0.021, HR = 1.292, 95%CI 1.040 - 1.604) were included in the nomogram (Figure 2). The nomogram indicated that age≥60, large NPTM (*n* = 2 or 3), large tumor size and advanced pTNM stage were the poor prognostic factors, but pTNM stage was still a most powerful one. The result of the nomogram was analogous to those of above-mentioned multivariate analysis. The calibration curve of nomogram showed that the predictive probability of 3-year survival was closely to the actual 3-year survival (Figure 3).

Subsequently, we compared the predictive accuracy of prognosis between the nomogram and TNM staging system (only pTNM stage). The C-index of nomogram was 0.718, compared with 0.689 of TNM staging system. The difference between nomogram and TNM staging system was significant (*P* < 0.001).

### Validation of preoperative serum tumor markers in an independent cohort

**Figure 4:**
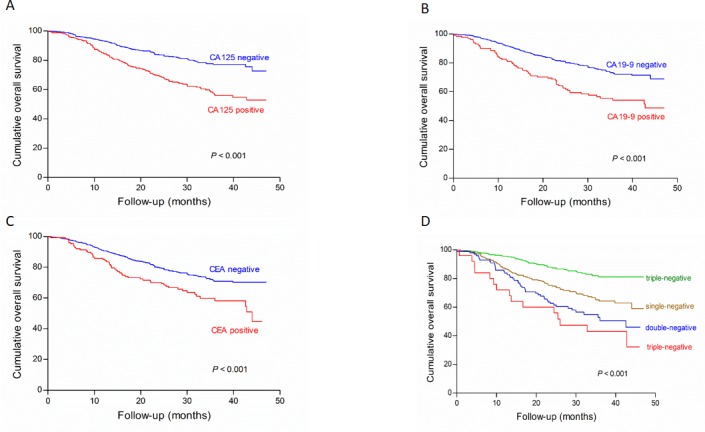
Survival analysis of subgroups of CA125 (A), CA19-9 (B), CEA (C) and their combined detection (D) in the validation cohort There were significant differences on survival outcomes between positive and negative subgroups in CA125, CA19-9, CEA and their combined detection, respectively (all *P* < 0.001)

Six hundred and fifty patients (650/668, 97.3%) were followed up with median survival time of 30.9 (0.5-47.0) months in the validation cohort. For all 650 patients, the 1- and 2- cumulative overall survival rates were 78% and 69%, respectively. The negative groups of all three tumor markers had significantly higher survival rates than the positive groups, respectively (Figure 4A, 4B, 4C, all *P* < 0.001). In univariate survival analysis, tumor location, tumor size, macroscopic type, differentiation grade, pT stage, pN stage, M stage and pTNM stage were significantly associated with prognosis (all *P* < 0.05). In a multivariate analysis, tumor size (*P* = 0.005), macroscopic type (*P* = 0.013), pTNM stage (*P* < 0.001), CA 125 (*P* = 0.005) and CA 19-9 (*P* = 0.023) were independent prognostic factors.

**Figure 5:**
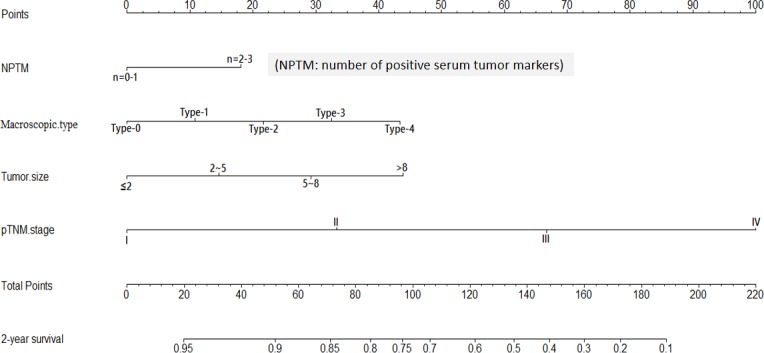
Nomogram of NPTM and clinicopathological traits in the validation cohort

**Figure 6:**
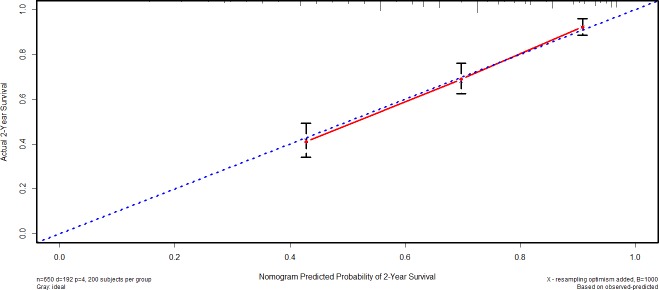
Calibration curve of nomogram in the validation cohort

By using Cox regression test, macroscopic type (*P* = 0.013), tumor size (*P* = 0.003), pTNM stage (*P* < 0.001) and NPTM (*P* = 0.011, HR = 1.3502, 95%CI 1.099 - 2.052) were included in the nomogram (Figure 5). The result of the nomogram was analogous to those of above-mentioned multivariate analysis. The calibration curve of nomogram showed that the predictive probability of 2-year survival was closely to the actual 2-year survival (Figure 6). The C-index of nomogram was 0.747, compared with 0.694 of TNM staging system. The difference between nomogram and TNM staging system was significant (*P* < 0.001).

## DISCUSSION

Serum tumor markers are quite commonly detected before and after surgery for most kinds of tumors. Finding a specific tumor marker will help to increase the accuracy of diagnosis and predict the prognosis of GC patients, however, the role of existing tumor markers in GC is still controversial. In addition to some routinely applied tumor markers, such as α-Fetoprotein (AFP), CEA, CA19-9 and carbohydrate antigen 72-4 (CA72-4), there are still many other tumor markers used in different hospitals [16]. According to the previous studies, those serum tumor markers showed poor sensitivity and specificity of diagnosis. Nevertheless, the prognostic value of serum tumor markers was supported by many previous studies.

All enrolled patients in our study were pathologically diagnosed as GC, and our purpose was to explore the prognostic value of preoperative serum tumor markers in GC patients, therefore, the cut-off values of three tumor markers were recalculated. The cut-off values of tumor markers in previous studies were defined by the manufacturer recommendation or calculated by the receiver operating characteristic curve. X-tile plot was applied in our study as it is a time-dependent cut-off value analysis based on the survival information. The new cut-off values of CA125, CA19-9 and CEA were 13.72 U/ml, 23.36 U/ml and 4.28 ng/ml, respectively. The positive rate of CA19-9 was reported from 17.1% to 55.8%, and CEA from 16.1% to 46.6% [15, 17–21], and the positive rates of these two markers in this study were within these ranges (CA19-9: 20.0%, CEA: 19.2%). The positive rate of CA125 was 42.3%, which was much higher than other studies with the range between 21% and 31.1%. This big difference might due to that the recalculated cut-off value of CA125 in our study was obviously lower than that of other studies (20 - 35 U/ml) [12, 22, 23].

In a previous study by Erdal et al, which enrolled 106 GC patients, the result showed that there was no significant correlation between pT stage and pN stage and serum CA125, CA 19-9 and CEA levels. Moreover, only elevated CEA was significantly associated with M stage [24]. We also analyzed the association between preoperative serum tumor markers and clinicopathological traits, and found that pT stage, pN stage, M stage and pTNM stage were significantly correlated with the positivity of three tumor markers, which was consistent with previous studies [17, 25]. Our result indicated that GC patients with positive tumor markers might suffer from more advanced pathological stage. When three tumor markers were combined, the proportion of stage III-IV and lymph node metastasis in triple-positive group was higher than the proportion in the positive group of each single tumor marker, which was similar to the previous report [26]. The result indicated that combined detection of serum tumor markers could help clinician to make a more accurate preoperative staging. The tumor size larger than 5 cm might reflect in high possibility of tumor marker positivity (all *P* < 0.001), which resembled the previous study [27]. The similar result appeared in macroscopic type that positive tumor markers might indicate more advanced macroscopic type.

The three tumor markers exhibited difference in gender, age, tumor location and differentiation grade. The positive CA125 and CA19-9 were more likely to be seen in female patients, while positive CEA was more trend to be seen in male patients. The positive rates of CA125 and CA19-9 were higher in tumors occupying whole stomach than in tumors occupying limited one part of stomach. Our result was in agreement with previous studies that the sites of GC would affect serum marker levels [22, 28], and the reason might be that tumors occupying whole stomach were generally correlated with large tumor size and advanced pathological stage. The positive rates of tumor markers among the GC patients with different differentiation grade were not statistically significant in some previous studies [22, 29]. However, Seok et al reported that positive CEA were related to the grade of differentiation [30], and the present study found that positive CA125 was significantly correlated with differentiation grade. In summary, the differentiation grade has little impact on GC marker levels. In a multivariate analysis by logistic regression, we confirmed that pT stage, pN stage and gender were independent correlated factors of positive CA125 and CEA, while pT stage, pN stage and age were independent correlated factors of positive CA19-9.

In our study, patients in the positive groups of CA125, CA19-9 or CEA had significantly poorer overall survival than patients with negative marker level, which was similar to the previous researches [17, 19]. When three tumor markers were combined, the increasing number of positive tumor markers might indicate a worse overall survival (Figures 1D and 4D). The possible reason might be that tumor markers as prognostic factors showed superimposed effect. In addition to tumor markers, our result showed that clinicopathological traits, including age, tumor location, tumor size, macroscopic type, differentiation grade and pTNM stage, were significantly associated with overall survival. Multivariate analysis showed that tumor size, pTNM stage and CA125 were independent prognostic factors of GC patients. Zhou et al reported that CA125 was an independent prognostic factor of GC patients, which was analogous to our study [31]. However, Liu et al and Tocchi et al found that both CA19-9 and CEA provided independent predictive value in gastric cancer patients [19, 32], and Chen et al found that only CEA was an independent prognostic factor. Moreover, some studies even considered that tumor marker could not be an independent prognostic factor [33]. What cause the difference between our results and these studies might be discrepancies in the number of patients, the heterogeneity of enrolled patients, the detection technique and cut-off values.

Nomogram is an intuitionistic and widely applied method to predict the prognosis of individual patient on the basis of some valuable parameters. In our study, we figured out that the nomogram visually showed the impact of some clinicopathological traits on the prognosis of GC patients in both training and validation cohorts. In the light of nomogram, the prognosis of individual patient could be well predicted. In order to maximize the use of serum tumor markers, NPTM was proposed and included in nomogram *via* a stepwise algorithm. The predictive accuracy of nomogram was well illustrated through calibration curve. Moreover, we compared the predictive accuracy between nomogram and TNM staging system, and the result showed that nomogram with NPTM and other parameters was better than pTNM stage alone. According to the nomogram, however, we still regarded pTNM stage as one of the most important parameter in GC, but more importantly, other indexes like NPTM and tumor size should also be noticed.

There were some limitations to our study. Firstly, this is a retrospective analysis comes from a single center in western China, the results of this study may not represent overall Chinese population well. Secondly, our study did not take in the control groups including health person and patients with benign lesions, and the comparison of serum tumor marker levels in different groups could not be made. Thirdly, CA72-4 and CA15-3 were not routinely tested for GC patients in our hospital, therefore they were not analyzed and discussed in this study.

In conclusion, preoperative levels of serum CA125, CA19-9 and CEA were correlated with most of clinicopathological traits, especially pTNM stage. The positivity of CA125, CA19-9 and CEA could provide important prognostic information in GC patients and indicated less favorable outcomes. In addition, CA125 was an independent prognostic factor for GC patients and could be further investigated. Nomogram based on NPTM could accurately predict the prognosis of GC patients.

## MATERIALS AND METHODS

The West China Hospital research ethics committee approved retrospective analysis of anonymous data. Signed patient informed consent was waived per the committee approval, because it was a retrospective analysis.

### Patients

We retrospectively enrolled patients who were diagnosed with resectable primary GC and underwent gastrectomy from January 2005 to December 2013 in the Department of Gastrointestinal Surgery, West China Hospital, Sichuan University. Patients from January 2005 to December 2011 were enrolled into the training cohort, and patients form January 2012 to December 2013 were enrolled into the validation cohort. Patients with neoadjuvant chemotherapy and incomplete clinical data were excluded from the study.

Clinicopathological traits including tumor location, tumor size, microscopic type, differentiation grade, pathological T stage (pT stage), pathological N stage (pN stage), M stage and pathological TNM stage (pTNM stage) were collected according to the AJCC 7^th^ edition. In order to analyze the correlation between serum tumor markers and pN stage, N_+_ stood for the set of N1, N2 and N3.

All enrolled patients received regular follow-up. Outpatient visit was considered to be the optimal choice, meanwhile, telephones and mails were adopted as two main supplementary follow-up methods. According to the National Comprehensive Cancer Network (NCCN) gastric cancer guideline, follow-up was carried out every 3 to 6 months for 1 to 2 years, every 6 to 12 months for next 3 to 5 years, and annually thereafter. The follow-up time of patients in both training and validation cohorts was up to January 2016. The loss of follow-up was mainly due to the change of phone number or home address.

### Cut-off value

The three serum tumor markers were measured within one week before surgery at the clinical laboratory of West China Hospital through Elecsys Modular E170 (Roche Diagnostics, Switzerland). The clinical reference cut-off values of CA125, CA19-9 and CEA were 35 U/ml, 22 U/ml and 3.4 ng/ml, respectively. Since there was no healthy person (defined as person without GC and other malignancies) as control in this study, we recalculated the cut-off values through X-tile plot, and the recalculated cut-off values of CA125, CA19-9 and CEA were 13.72 U/ml, 23.36 U/ml and 4.28 ng/ml, respectively. Patients were divided into positive (+) group and negative (−) group for each marker according to the recalculated cut-off values. Patients with tumor marker levels lower than cut-off values were distributed into negative group, and other patients with tumor marker levels higher than cut-off values were distributed into positive group. Using combined detection, patients were divided into four groups: negative group, single-positive group, double-positive group and triple-positive group.

### Statistics

The statistical analysis was performed using SPSS 22.0 statistical software (SPSS Inc., Chicago, IL, USA). The X-tile plot (Version 3.6.1, Yale University) was used to calculate the cut-off values of serum tumor markers. The χ^2^, rank sum and Student's T tests were used to evaluate the association between tumor markers and clinicopathological traits for univariate analysis and logistic regression test for multivariate analysis. Kaplan-Meier method and Cox regression test were used to analyze univariate and multivariate prognostic factors, respectively. Nomogram was used to analyze the prognostic value of tumor markers and clinicopathological traits through R for Windows (Version 3.2.0, R Foundation for Statistical Computing). Two-tailed *P* value less than 0.05 was considered as statistically significance.
